# OmpW overexpression in meropenem-exposed *Acinetobacter baumannii* persister cells enhances virulence and reveals a candidate target

**DOI:** 10.3389/fmicb.2025.1656485

**Published:** 2025-09-15

**Authors:** Bin Liu, Junan Wang, Jincheng Hu, Liang Li, Lei Liu

**Affiliations:** ^1^Respiratory and Critical Care Medicine Department, The First Affiliated Hospital, Hainan Medical University, Haikou, Hainan, China; ^2^Hainan Province Clinical Medical Center of Respiratory Disease, Haikou, Hainan, China

**Keywords:** *Acinetobacter baumannii*, outer membrane protein W, persister cells, meropenem, resistance

## Abstract

**Objective:**

*Acinetobacter baumannii* is one of the most drug-resistant microorganisms in hospital-acquired infections. The treatment of choice for *A. baumannii* infections is carbapenems (e.g., meropenem). However, *A. baumannii* can develop resistance to all clinical antibiotics associated with the formation of persister cells. We first assessed the virulence and differential expression levels of outer membrane protein W (OmpW) in *A. baumannii* persister cells and *A. baumannii* regular cells *in vivo* and *in vitro* after exposure to meropenem.

**Methods:**

Persister cells were confirmed using a standard method. OmpW characterization was performed using western blot analysis, and OmpW expression was detected using real-time polymerase chain reaction (PCR) after ribonucleic acid (RNA) extraction. An *A. baumannii* virulence assay was performed using the *Galleria mellonella* larvae model. Relative expression was calculated using the 2^−ΔΔCT^ method.

**Results:**

The presence of bona fide *A. baumannii* persister cells was confirmed after 48 h of meropenem exposure at 15 μg/mL, with levels reaching 0.3216% of the initial bacterial population and a survival fraction of 0.081%. OmpW genes were highly expressed at more than 2.68-fold (*p* = 0.01) with meropenem exposure at 1 μg/mL and 8.61-fold (*p* = 0.0005) with meropenem exposure at 15 μg/mL. There was a significant difference in the lethal dose 50% (LD_50_) at 24 h postinfection between persister cells (2.01 × 10^5^ CFU/larva) and regular cells (4.73 × 10^5^ CFU/larva) at *p* < 0.05. Similarly, there was a significant difference between the LD_50_ at 48 h for persister cells (1.61 × 10^5^ CFU/larva) and regular cells (4.08 × 10^5^ CFU/larva) at *p* < 0.05. However, there was no statistically significant difference in the LD_50_ at 72, 96, and 120 h postinfection.

**Conclusion:**

OmpW overexpression in meropenem-exposed *A. baumannii* persister cells enhances virulence and reveals a candidate target for preventing and controlling *A. baumannii* persister cells.

## Introduction

*Acinetobacter baumannii* is one of the most prevalent and drug-resistant microorganisms behind a variety of hospital-acquired infections contracted in intensive care units, including ventilator-associated pneumonia and hospital-acquired pneumonia (Liu and Liu, 2021; Latifi et al., 2023). The treatment of choice for *A. baumannii* infections is carbapenems, such as meropenem (Liu and Liu, 2021). The mortality rate for infections caused by drug-resistant *A. baumannii* is ~75% (Latifi et al., 2023).

*A. baumannii* can develop resistance to clinical antibiotics and survive for a long time in disinfected hospital environments, which is primarily attributed to antibiotic resistance and the formation of persister cells (Latifi et al., 2023; Catel-Ferreira et al., 2016; Schmitt et al., 2023). The latest research findings show that notwithstanding their susceptibility to antibiotics, persister cells of *A. baumannii* can survive high concentrations of antibiotics, which indicates that these persister cells may be involved in the evolution of antibiotic resistance and may be one of the primary reasons for the failure of antibiotic therapy (Schmitt et al., 2023). The formation of *A. baumannii* persister cells is also responsible for treatment failure in biofilm-associated infections (Latifi et al., 2023; Tseng et al., 2016). Furthermore, the findings of a particular study indicate that high levels of outer membrane protein W (OmpW) expression are linked to high levels of virulence in *A. baumannii* persister cells (Schmitt et al., 2023). However, another study proposes that the clinical impact of the formation of *A. baumannii* persister cells on treatments targeting infections is still unclear, and further studies are still needed (Chung et al., 2017). Considering the fact that the formation of persister cells is closely linked to antibiotic tolerance, antibiotic resistance, failure of antibiotic therapy, and the relapse of infections (Kashyap et al., 2022), it is imperative to explore effective antibiotic combinations that target persister cells in *A. baumannii* infections. Some studies have revealed that the synergistic bactericidal effect of drug combinations, including colistin and amikacin or polymyxin B and meropenem, effectively eradicates *A. baumannii* persister cells (Chung and Ko, 2019; Gallo et al., 2017).

Presently, the putative mechanism responsible for tolerance and resistance to high concentrations of meropenem exhibited by *A. baumannii* persister cells has not been fully or clearly explained. A study by Schmitt et al. proposes that OmpW, as part of the efflux pump apparatus, is linked to *A. baumannii* persister cell formation (Catel-Ferreira et al., 2016; Schmitt et al., 2023). Due to the importance of OmpW in pathogenesis, the formation of OmpW channels for the uptake of small molecules and the regulation of OmpW expression appear to be essential for the adaptive response of *A. baumannii* to conditions of antibiotic stress (Schmitt et al., 2023; Hong et al., 2006).

We hypothesize that OmpW plays a key role in the survival of *A. baumannii* after exposure to high concentrations of meropenem; however, the mechanism underpinning the role of OmpW still needs to be elucidated. In this study, we first assess the virulence and then the differential expression levels of OmpW in *A. baumannii* persister cells and *A. baumannii* regular cells *in vivo* and *in vitro* after exposure to meropenem.

## Methods

### *A. baumannii* strains, culture conditions, and antimicrobial susceptibility

The experiments were carried out using the *A. baumannii* regular cells, which was previously isolated from tracheal aspirates of *A. baumannii* pneumonia patient by the Respiratory and Critical Care Medicine Department and Microbial Department in our Hospital following the protocol approved by the Ethics Committee in Research.

*A. baumannii* regular cells from clinical strains with sensitivity to meropenem were used in this experiment. The conditions under which *A. baumannii* persister cells formed were consistent with the literature (Schmitt et al., 2023). The bacteria strains were stored at −80 °C in brain heart infusion broth with 20% glycerol. All experiments were conducted at 37 °C in Luria-Bertani broth with shaking at 180 rpm.

The susceptibility of *A. baumannii* strains to antimicrobial agents was determined using a microdilution method in accordance with the guidelines of the Clinical and Laboratory Standards Institute. The agents tested included imipenem, meropenem, levofloxacin, ceftazidime, cefepime, piperacillin/tazobactam, cefoperazone/sulbactam, tigecycline, colistin.

### Characterization of OmpW using western blot analysis

Following the method in the literature (Bista et al., 2023), fractions of eluted OmpW were characterized using western blotting, and 20 μg of total protein was separated from the OmpW samples using sodium dodecyl sulfate-polyacrylamide gel electrophoresis. The OmpW samples were electrotransferred onto polyvinylidene fluoride (PVDF) membranes (Bio-Rad Laboratories, Hercules, CA, USA, 162-0182). The PVDF membranes were blocked with 3% bovine serum albumin in phosphate-buffered saline (PBS) supplemented with 0.05% Tween 20 (PBS-T) for 1 h at room temperature, followed by incubation with primary antibodies at 1:1,000 dilution and room temperature for 2 h. All antibodies used in this study, including anti-OmpW antibodies, rabbit polyclonal anti-42-kDa OmpW antibodies, anti-leukotoxin rabbit polyclonal antibodies, and anti-leukotoxin mouse monoclonal antibodies, were procured from Cocalico Biologicals Inc (Stevens, PA, USA) or Lampire Biologicals (Pipersville, PA, USA). The PVDF membranes were incubated with goat anti-rabbit IgG, alkaline phosphatase conjugate secondary antibodies (MilliporeSigma, Burlington, MA, USA, 12-448) at 1:4,000 dilution and room temperature for 1 h after washing with PBS-T. Premixed 5-bromo-4-chloro-3-indolyl phosphate/nitro blue tetrazolium (BCIP/NBT; MilliporeSigma, Burlington, MA, USA, B6404), which is a chemiluminescence detection solution, was used to visualize protein signals.

### Persister cell assay

A persister cell assay was implemented in triplicate in accordance with the protocol described by Schmitt et al. (2023). A colony of the *A. baumannii* regular cells was inoculated and grown overnight in Lysogeny broth (LB) at 37 °C for over 16 h. This culture was diluted at 1:50 in 12 mL fresh LB and incubated at 37 °C until the mid-exponential growth phase. Aliquots of the culture were collected for ribonucleic acid (RNA) extraction and determination of the concentration of colony-forming units (CFU) in CFU/mL before the addition of meropenem (Hanhui Pharmaceutical Co. Ltd., Hangzhou, Zhejiang, China). The culture was diluted up to 10^−6^, and each 10 μL dilution of the culture after spotting on nutrient agar was incubated at 37 °C for 24 h. The initial cell density was determined after the aliquots were collected, and RNA purification was performed. Meropenem at 1 μg/mL was the minimum concentration required to inhibit visible growth of regular cells under standard laboratory conditions. Meropenem at 15 μg/mL was the supra-lethal dose required to select persister cells and study their unique adaptations, antibiotic tolerance and virulences under standard laboratory conditions. The remaining culture was exposed to meropenem at 1 μg/mL (1-fold minimum inhibitory concentration) and 15 μg/mL (15-fold minimum inhibitory concentration) for 48 h at room temperature. Then, 1 mL aliquots were collected to determine the persister cell fractions and for RNA extraction after 12, 24, and 48 h of meropenem exposure at 1 and 15 μg/mL, respectively. The aliquots were centrifuged at 9,660 × *g* for 7 min and then washed with 0.85% sterile saline solution to remove potential meropenem residues. Each 10 μL dilution of the culture was spotted on nutrient agar after preparing a serial decimal dilution. The persister cell fractions were determined using the CFU concentration (CFU/mL) of surviving cells after incubation at 37 °C for 24 h. The resistance phenotype was determined by identifying the minimum inhibitory concentration of the two *A. baumannii* strains (1 μg/mL with meropenem) after persistence assays. An *A. baumannii* regular cells culture was also grown—as a control group—under the same time and temperature conditions without the adjunction of meropenem, and the CFU concentration was determined after 12, 24, and 48 h.

### Virulence assay in the *Galleria mellonella* larvae model

Virulence assays of *A. baumannii* persister cells and *A. baumannii* regular cells were implemented in the *Galleria mellonella* larvae model in accordance with the protocol described by Schmitt et al. (2023). The experiments were executed in triplicate. *G. mellonella* larvae were fed a diet consisting of several flours and honey at 28 °C in the laboratory, and *G. mellonella* larvae in the final instar stage weighing 240–260 mg were used for experimentation in this study. The *G. mellonella* larvae were injected in the hemocoel via the last right proleg with a 10 μL of *A. baumannii* suspension (10^2^-10^7^ CFU/larva) in PBS 1 × pH 7.4 or only PBS (control group). The *G. mellonella* larvae were then incubated at 37 °C for 120 h. A survival assay of infected *G. mellonella* larvae was performed based on response to touch stimuli every 24 h. The LD_50_ was analyzed at specific time points after infection with *A. baumannii*.

### Quantitative real-time polymerase chain reaction (qRT-PCR)

**Figure 1:**
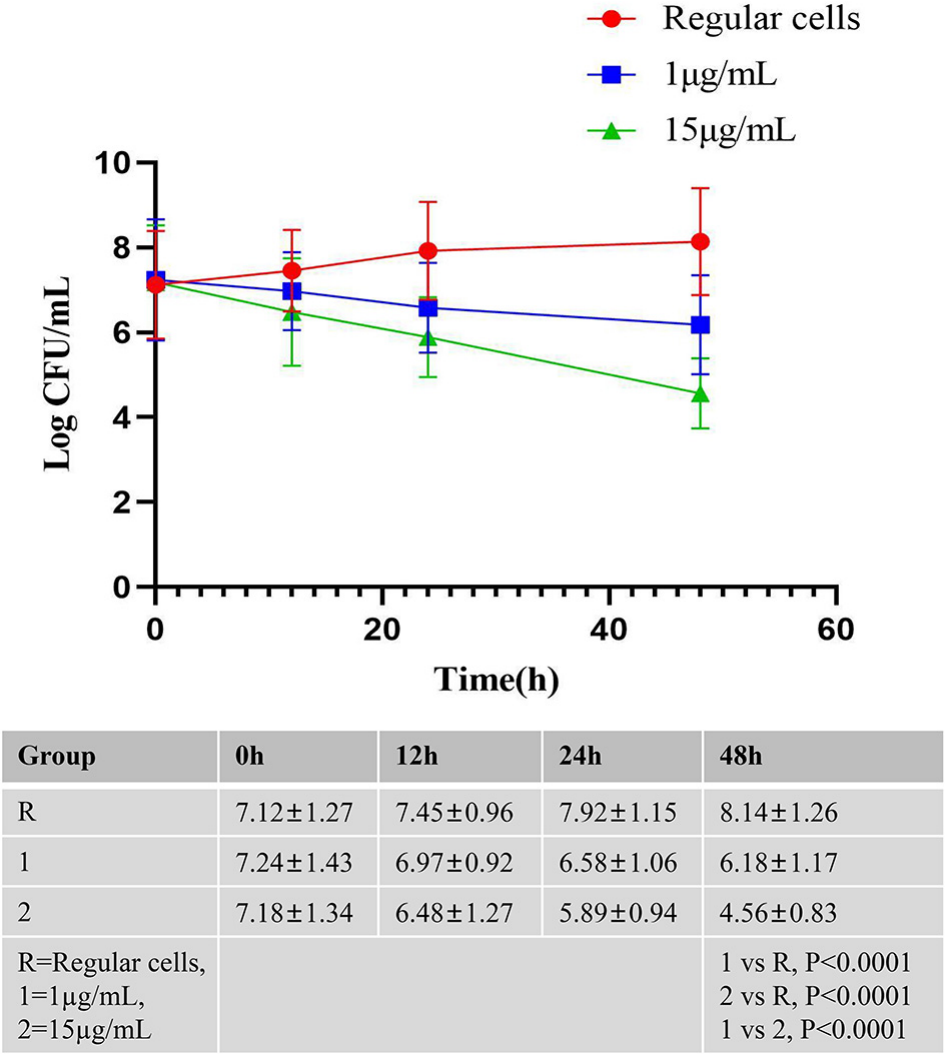
The killing curves of *A. baumannii* Regular cells after 48 h exposure to meropenem. *A. baumannii* Regular cells was cultured until the middle exponential phase and exposed to meropenem at 1 and 15 μg/mL, in triplicate, the number of CFU/mL were determined after 12, 24, and 48 h. *A. baumannii* Regular cells culture as a control group was also grown at the same conditions of time and temperature without the adjunction of meropenem, in triplicate, the number of CFU/mL were determined after 12, 24, and 48 h.

**Figure 2:**
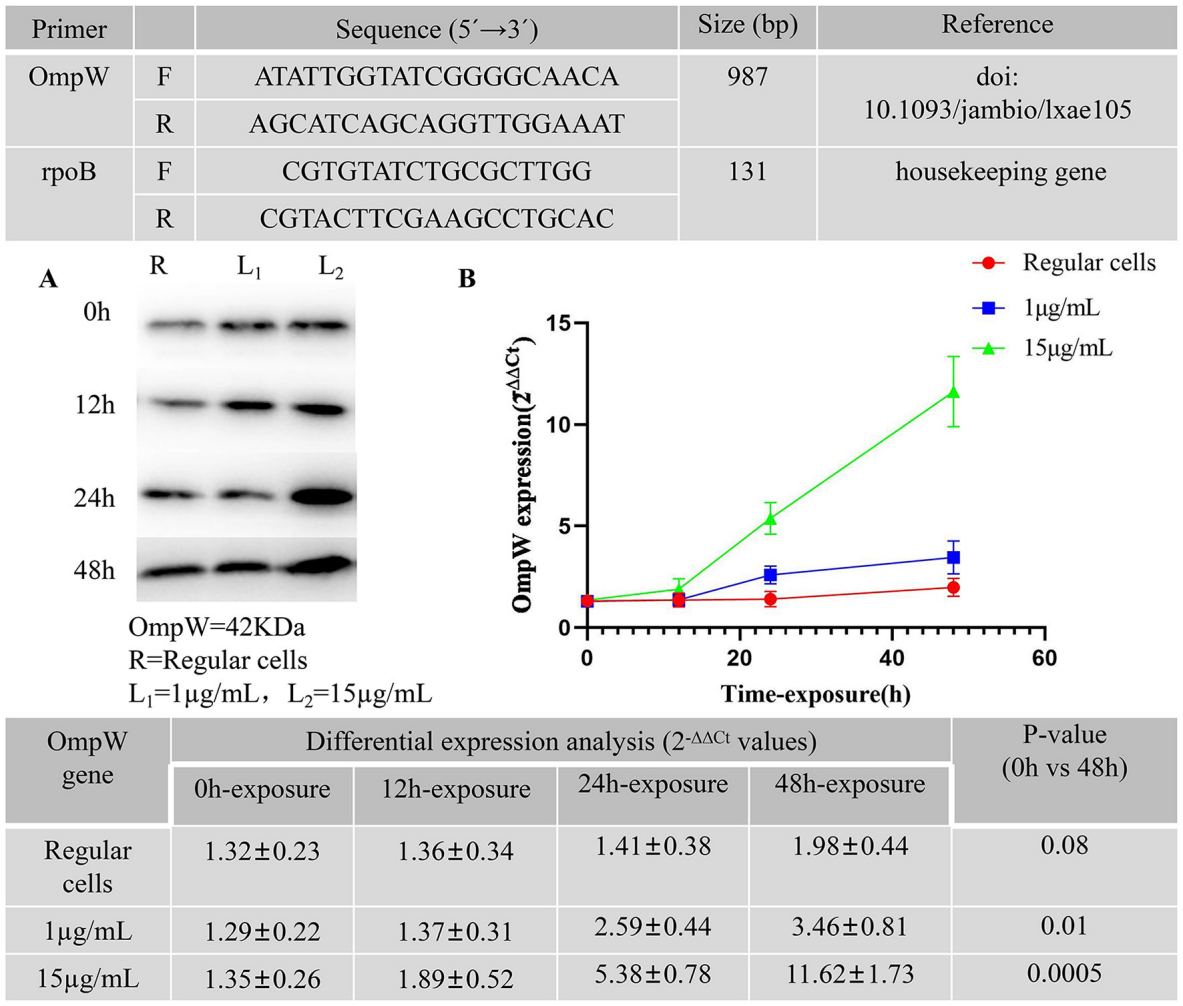
Primers used in the study for qPCR. **(A)** Western blot analysis of OmpW after meropenem exposure at 1 and 15 μg/mL up to 48 h in *A. baumannii* persister cells. *A. baumannii* Regular cells as a control group was analyzed. **(B)** Relative expression levels of OmpW after meropenem exposure at 1 and 15 μg/mL, in triplicate, up to 48 h in *A. baumannii* persister cells. *A. baumannii* Regular cells as a control group was analyzed.

Quantitative real-time polymerase chain reaction (qRT-PCR) was performed following the protocol outlined by Schmitt et al. (2023) and Nayak et al. (2024). The relative OmpW expression levels from *A. baumannii* were evaluated using 1 μg of purified total RNA as a template for reverse transcription, which was implemented using a high-capacity cDNA reverse transcription kit (Roche, Germany) following the manufacturer's instructions. Polymerase chain reaction (PCR) amplifications were performed in a Rotor-Gene Q (Qiagen, Hilden, Germany) real-time PCR system using the BioFACT 2X real-time PCR master mix (BIOFACT, Co Ltd, Yuseong-Gu, South Korea). The specific primers used for real-time PCR are listed in Figure 1. Relative expression was calculated using the 2^−Δ*ΔCT*^ method, and RNA input was normalized against the rpoB housekeeping gene (Figure 2). Gene expression levels were determined as the fold change relative to the transcriptional level of each corresponding gene in the *A. baumannii* regular cells. All reactions were carried out in duplicate, and the experiments were repeated three times.

### RNA extraction

The RNA extraction was performed according to the protocol outlined by Schmitt et al. (2023) and Nayak et al. (2024). Whole RNA was extracted using TRIzol LS reagent (Invitrogen, MA, USA) according to the manufacturer's instructions. To extract RNA, centrifugation of the aliquots at 8,000 × *g* was performed twice for 5 min, followed by washing with 0.85% sterile saline solution, and then resuspension in 250 μL of sterile Milli-Q water. After 750 μL of TRIzol was added to the aliquots, the samples were subsequently allowed to incubate at room temperature for 5 min. After adding 200 μL of chloroform, the samples were allowed to incubate at room temperature for 10 min. Subsequently, the samples were centrifuged at 12,000 × *g* for 15 min at 4 °C, followed by the addition of 1 mL of 75% ethanol into the RNA pellet, centrifugation at 7,500 × *g* for 5 min at 4 °C, and air-drying of the precipitated RNA pellet for 10 min. The precipitated RNA was resuspended in 20 μL RNase-free water (Invitrogen) and stored at −80 °C. A NanoDrop One spectrophotometer (Thermo Scientific, MA, USA) was used to determine the total RNA concentration, and RNase-free DNase (Invitrogen) was used to detect the total RNA in the samples.

### Statistical methods

Data from the *in vivo* experiments were analyzed using Kaplan–Meier survival curves in GraphPad Prism 9.0 (GraphPad Software Inc, La Jolla, CA, USA). The LD_50_ value and the respective 95% confidence intervals (95% CI) for *A. baumannii* regular cells and *A. baumannii* persister cells were analyzed using version 23 of IBM SPSS Statistics software (IBM Corporation, Armonk, NY, USA) by reading the assessed cell number at a probability of 0.5 and fitting the data to a logit regression model. Relative median potency estimates were used to identify the difference between the *A. baumannii* regular cells and *A. baumannii* persister cell samples. If the relative mean potency estimate of the 95% confidence interval does not equal 1 on the untransformed scale, the LD_50_ could subsequently be regarded as significantly different. GraphPad Prism was also used to analyze the experimental data, which are presented as the mean ± standard deviation (SD). The descriptive statistics of the data were analyzed, and a two-way analysis of variance (ANOVA) for independent samples was performed, followed by a Tukey test to determine the differences for each variable. *P* < 0.05 was considered a statistically significant difference.

## Results

Susceptibility testing demonstrated that *A. baumannii* regular cells were resistant to most antibiotics except sensitive to meropenem, imipenem, tigecycline and colistin, and intermediate to cefoperazone/sulbactam (Table 1). Susceptibility testing demonstrated that *A. baumannii* persister cells were resistant to most antibiotics except intermediate to tigecycline and colistin (Table 1).

**Table 1:** Susceptibility profiles of nine *A. baumannii* strains examined in this study.

**Antibiotics**	**Strains (MIC, mg/L)**	
	**Regular cells**	**Persister cells**
Cefepime	>32 (R)	>32 (R)
Ceftazidime	>64 (R)	>64 (R)
Meropenem	0.25 (S)	>16 (R)
Imipenem	0.25 (S)	>16 (R)
Levofloxacin	>8 (R)	>8 (R)
Piperacillin/Tazobactam	>128 (R)	>128 (R)
Cefoperazone/Sulbactam	32 (I)	>64 (R)
Tigecycline	2 (S)	2 (S)
Colistin	1 (S)	0.5 (S)

R, resistant; I, intermediate; S, susceptible.

MIC, Minimum Inhibitory Concentration.

The killing curves for *A. baumannii* regular cells after 48 h of exposure to meropenem are presented in Figure 1. *A. baumannii* regular cells was cultured until the middle exponential phase and then exposed to meropenem at 1 and 15 μg/mL in triplicate. The CFU concentration was determined after 12, 24, and 48 h. An *A. baumannii* regular cells culture, as a control group, was also grown in triplicate under the same time and temperature conditions without the adjunction of meropenem, with the CFU concentration determined after 12, 24, and 48 h. The research results indicate the presence of bona fide *A. baumannii* persister cells after 48 h of exposure to meropenem at 15 μg/mL, with levels reaching 0.3216% of the initial bacterial population. Similarly, the survival fraction was demonstrated to be 0.081% when comparing the number of surviving cells exposed to meropenem at 15 μg/mL after 48 h to the cells in the *A. baumannii* regular cells culture.

The primers used for the quantitative PCR (qPCR) in this study are presented in Figure 2. The western blot analysis of OmpW in *A. baumannii* persister cells after meropenem exposure at 1 and 15 μg/mL for up to 48 h is presented in Figure 2A. The *A. baumannii* regular cells culture was analyzed as a control group. Figure 2B presents the relative OmpW expression levels in *A. baumannii* persister cells after meropenem exposure at 1 and 15 μg/mL in triplicate for up to 48 h. *A. baumannii* regular cells were also analyzed as a control group. The *x*-axis of Figure 2B shows the time points for meropenem exposure, while the *y*-axis tracks the fold difference of each gene to the threshold cycle (Ct) values. The differential expression of OmpW in response to meropenem exposure at 1 and 15 μg/mL for up to 12, 24, and 48 h was assessed. Our findings indicate that there were significant differences in the differential expression levels of the OmpW genes between the 0 and 48 h time points following meropenem exposure, as determined using ANOVA and Tukey's multiple comparison test. The OmpW genes were highly expressed by more than 2.68-fold (*p* = 0.01) with meropenem exposure at 1 μg/mL, and 8.61-fold (*p* = 0.0005) with meropenem exposure at 15 μg/mL.

**Figure 3:**
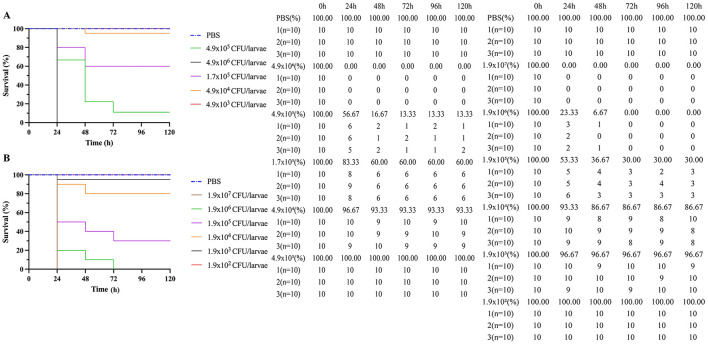
Kaplan-Meier survival curves of Galleria mellonella larvae inoculated with *A. baumannii* strain, in triplicate with ten larvae in each group. **(A)** Survival curves of *A. baumannii* Regular cells and **(B)** Survival curves of *A. baumannii* persister cells after exposure at 15 μg/mL to meropenem. Vehicle group (phosphate-buffered saline, PBS) was used as sterility and injection control.

As seen in Figure 3, *A. baumannii* persister cells and *A. baumannii* regular cells were injected in different quantities into *G. mellonella* larvae, and their survival rates were measured. The Kaplan–Meier survival curves for *G. mellonella* larvae inoculated with the *A. baumannii* strain in triplicate, with 10 larvae in each group, are also displayed in Figure 3. Figure 3A shows the survival curves for *A. baumannii* regular cells, and Figure 3B shows the survival curves for *A. baumannii* persister cells after exposure to meropenem at 15 μg/mL. The control group was used as the sterility and PBS injection. We found that *A. baumannii* persister cells are statistically significantly more lethal than *A. baumannii* regular cells to *G. mellonella* larvae (*p* < 0.05).

**Figure 4:**
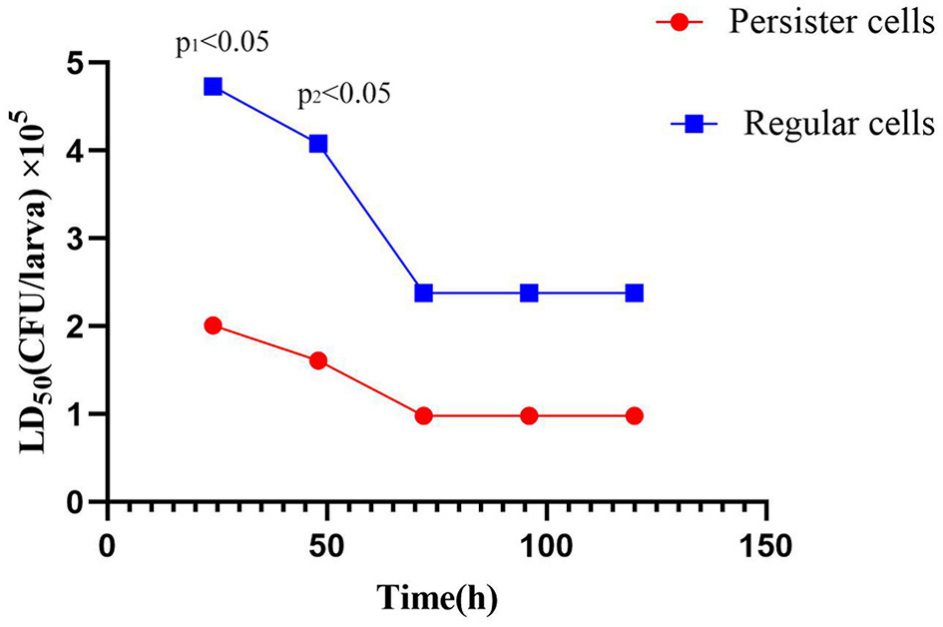
Comparison of LD_50_ values obtained by *A. baumannii* Regular cells and *A. baumannii* persister cells (post-exposure with 15 μg/mL meropenem) in *Galleria mellonella* larvae at different times of infection.

The LD_50_ values obtained for *A. baumannii* regular cells and *A. baumannii* persister cells (post-exposure to 15 μg/mL meropenem) in *G. mellonella* larvae at different times after infection are presented in Figure 4 and Table 2. There was a statistically significant difference in the LD_50_ at 24 h postinfection between *A. baumannii* persister cells (2.01 × 10^5^ CFU/larva) and *A. baumannii* regular cells (4.73 × 10^5^ CFU/larva) at *p* < 0.05. Similarly, there was also a statistically significant difference in the LD_50_ at 48 h postinfection between *A. baumannii* persister cells (1.61 × 10^5^ CFU/larva) and *A. baumannii* regular cells (4.08 × 10^5^ CFU/larva) at *p* < 0.05. However, there were no statistically significant differences in the LD_50_ at 72, 96, and 120 h postinfection between *A. baumannii* persister cells (0.98 × 10^5^ CFU/larva) and *A. baumannii* regular cells (2.38 × 10^5^ CFU/larva).

**Table 2:** Comparison of LD_50_ values obtained by *A. baumannii* Regular cells and *A. baumannii* Persister cells (post-exposure with 15 μg/mL meropenem) in *Galleria mellonella* larvae at different times of infection.

**Time (h)**	***A. baumannii***	**LD_50_ (CFU/larva)**	**Significance**	**95% CI (CFU/larva)**	**Potency estimate (mean)**	**Potency estimates (confidence interval)**
24	Regular cells	4.73 × 10^5^	Yes	3.66 × 10^5^-8.89 × 10^5^	2.12	1.11-4.49
	Persister cells	2.01 × 10^5^		1.49 × 10^5^-3.62 × 10^5^	0.46	0.19-0.94
48	Regular cells	4.08 × 10^5^	Yes	3.66 × 10^5^-8.89 × 10^5^	2.02	1.10-4.51
	Persister cells	1.61 × 10^5^		1.44 × 10^5^-3.61 × 10^5^	0.45	0.20-0.91
72	Regular cells	2.38 × 10^5^	No	1.02 × 10^5^-5.14 × 10^5^	2.01	0.70-9.79
	Persister cells	0.98 × 10^5^		0.68 × 10^5^-2.22 × 10^5^	0.44	0.17-1.46
96	Regular cells	2.38 × 10^5^	No	0.96 × 10^5^-4.26 × 10^5^	2.49	0.77-22.78
	Persister cells	0.98 × 10^5^		0.32 × 10^5^-1.43 × 10^5^	0.35	0.05-1.40
120	Regular cells	2.38 × 10^5^	No	0.92 × 10^5^-4.25 × 10^5^	2.51	0.71-22.79
	Persister cells	0.98 × 10^5^		0.33 × 10^5^-1.46 × 10^5^	0.36	0.05-1.39

Furthermore, we found that the high expression of virulence in the OmpW genes may be linked to adaptation to meropenem exposure in *A. baumannii* persister cells. In addition, considering the observed LD_50_ values in the *in vivo* data, *A. baumannii* persister cells have markedly lower LD_50_ values than *A. baumannii* regular cells at 24 and 48 h during the early infection phase. Our findings also indicate that *A. baumannii* persister cells are markedly more lethal than *A. baumannii* regular cells.

## Discussion

Previous studies have shown that persister cells play a key role in the adaptation and resistance demonstrated by *A. baumannii* to antibiotics, which ensures its survival (Catel-Ferreira et al., 2016; Schmitt et al., 2023). Although some studies have attempted to elucidate the unique structural characteristics and the generative mechanism underpinning *A. baumannii* persister cells, the putative mechanism and structure exhibited by *A. baumannii* persister cells are still poorly understood (Catel-Ferreira et al., 2016; Schmitt et al., 2023). OmpW is a natural candidate in *A. baumannii* for regulation after exposure to antibiotics, which is typically attributed with directly processing the metabolic flux of soluble components between intracellular compartments and the extracellular environment and the periplasmic space (Schmitt et al., 2023; Tseng et al., 2016; Chung et al., 2017; Kashyap et al., 2022; Chung and Ko, 2019; Gallo et al., 2017; Hong et al., 2006; Bista et al., 2023; Nayak et al., 2024; Shahryari et al., 2021). Previous studies further indicate that OmpW, as a member of the small outer membrane protein family, plays an extremely important role in the formation and drug resistance of *A. baumannii* persister cells (Kashyap et al., 2022).

OmpW is a β-barrel protein composed of 8 β-folded chains, which forms a long and narrow hydrophobic channel that is involved in various bacterial resistance and pathogenesis mechanisms, including drug resistance, salt concentration tolerance, and the regulation of pathogenicity and bacterial virulence (Hong et al., 2006; Bista et al., 2023; Nayak et al., 2024; Shahryari et al., 2021). In particular, OmpW maintains cellular homeostasis under stress and is involved in transporting hydrophobic molecules across the outer membrane, which impacts the virulence factors necessary for *A. baumannii* pathogenicity (Hong et al., 2006; Shahryari et al., 2021). Furthermore, OmpW plays a critical role in bacterial nutrient uptake, such as bacterial iron acquisition, which impacts the treatment of bacterial infections (Hong et al., 2006; Shahryari et al., 2021). Interestingly, studies on *Escherichia coli* show that the minimum inhibitory concentration of several antibiotics decreases with OmpW knockout, indicating that OmpW may be a strategic protein employed by bacteria to counteract unfavorable environments and antibiotic exposure (Lin et al., 2010). Another study found that OmpW can combine with carbapenemases, which can elevate the carbapenemase levels in the periplasmic space and decrease antibiotic activity in the molecular targets of carbapenemases (Wu et al., 2016).

In this study, we first assessed the virulence and then the differential expression levels of OmpW in *A. baumannii* persister cells and *A. baumannii* regular cells *in vivo* and *in vitro* after exposure to meropenem. OmpW expression in *A. baumannii* persister cells markedly increased when exposed to high concentrations of meropenem, which leads to outcomes such as meropenem adaptation and resistance—similar to porins limiting the entry of β-lactams into cells (Schmitt et al., 2023; Nayak et al., 2024). Thereby, *A. baumannii* persister cells protect themselves from high concentrations of meropenem by increasing OmpW expression, which may facilitate limiting the intracellular transfer and concentration of meropenem. In addition, the data presented in our study showed that susceptibility testing demonstrated that *A. baumannii* regular cells were resistant to most antibiotics except sensitive to meropenem, imipenem, tigecycline and colistin, and intermediate to cefoperazone/sulbactam, however, persister cells were resistant to most antibiotics except sensitive to tigecycline and colistin. This may be explained by the presence of OmpW overexpression in *A. baumannii* strains, which typically indicate sensitivity to colistin, but is not affected to tigecycline.

OmpW has been revealed to be critical for the invasion and adherence of mammalian host cells (Gil-Marqués et al., 2022). OmpW overexpression plays a key role in the higher number of *G. mellonella* deaths caused by *A. baumannii* persister cells than by *A. baumannii* regular cells. Our research data on OmpW *in vivo* indicate that *A. baumannii* persister cells are more virulent than *A. baumannii* regular cells during the early stages of infection, as the data show significantly lower LD_50_ values for *A. baumannii* persister cells than for *A. baumannii* regular cells.

There were statistically significant differences in the LD_50_ of *A. baumannii* persister cells and *A. baumannii* regular cells at 24 and 48 h postinfection; however, there were no statistically significant differences in the LD_50_ at 72, 96, and 120 h postinfection. The high virulence expression of OmpW genes tends to reach stable levels after 72 h, with no significant difference between *A. baumannii* persister cells and *A. baumannii* regular cells, indicating that OmpW virulence has returned to its baseline level. Our research results are consistent with those in the literature (Schmitt et al., 2023), as high virulence expression while confronting challenging meropenem exposure is supposed to be transient. Our data on the LD_50_ values at 72, 96, and 120 h postinfection for *A. baumannii* persister cells and *A. baumannii* regular cells were not significantly different. This may be interpreted to mean that *A. baumannii* persister cells revert to the typical *A. baumannii* phenotype after severe stress ceases; furthermore, the high expression of virulence genes may be self-regulatable and transient depending on the antibiotic stress situation.

Previous studies have demonstrated that OmpW is a protein with high immunogenicity and a candidate for developing effective vaccines and therapies to prevent and treat *A. baumannii* infections (Huang et al., 2015). OmpW is indeed a potential target for the development of new therapeutic antibiotics for controlling *A. baumannii* infections. Our research findings elucidate the intrinsic usefulness of OmpW as a promising target for new therapeutic antibiotics and vaccines to prevent and control *A. baumannii* persister cells and *A. baumannii* regular cells.

We validated protein expression using Western blot, addressing the potential transcription-translation decoupling issue associated with relying solely on mRNA data. Schmitt et al. (2023) consistently reports OmpW as the more dominantly upregulated protein (10.5- to 13.7-fold) compared to OmpA (5.5- to 6.5-fold). The data unequivocally show that OmpW is more significantly overexpressed in persister cells. The authors even suggest OmpW's unique role in virulence and stress response, further highlighting its predominance. Our data also highlight that OmpW plays a predominant role in virulence regulation, providing new evidence for target prioritization.

This study also has limitation, it is well-established that meropenem challenge induces a complex adaptive response in *A. baumannii* beyond OmpW upregulation, the other mechanisms also possibly contributing to persister cells formation consist of upregulation of efflux pumps (e.g., AdeABC, AdeFGH), altered expression of other outer membrane porins (e.g., OmpA, CarO). Further research is still needed to clarify the role of the aforementioned mechanisms in *A. baumannii* persister cells. Another limitation in this study, while our study confirms and extends the findings of Schmitt et al. by demonstrating the nature of OmpW-mediated virulence enhancement and using a clinical strain from a pneumonia patient, we acknowledge that further phenotypic characterization of persister cells is needed to fully establish their antibiotic tolerance profile *in vivo*. Our current G. mellonella data demonstrate intrinsic virulence differences but do not directly probe antibiotic tolerance. Future studies incorporating this design could reveal whether OmpW-overexpressing persister cells maintain their survival advantage even when hosts receive meropenem treatment—a critical question for clinical translation.

Further research may contribute to elucidating the molecular mechanisms underpinning the contribution of OmpW overexpression to the formation of *A. baumannii* persister cells in situations where the *A. baumannii* cells are stressed by meropenem exposure. We propose that because of the potential ineffectiveness of antibiotic monotherapies, it is imperative to explore new infection control strategies to combat the formation of *A. baumannii* persister cells—including effective antibiotic combinations, new antibiotics targeting OmpW expression, and highly effective measures for preventing *A. baumannii* infection. It is essential to elucidate the pathogenesis of persister cell formation through future research, as well as the putative mechanism via which OmpW is regulated, which can also further facilitate the development of new therapeutic antibiotics and vaccines.

## Conclusion

In this study, we assessed the virulence and differential expression levels of OmpW in *A. baumannii* persister cells and *A. baumannii* regular cells *in vivo* and *in vitro* after exposure to meropenem. Our research data on OmpW *in vivo* indicate that *A. baumannii* persister cells are more virulent than *A. baumannii* regular cells during the early stages of infection because the data show significantly lower LD_50_ values for *A. baumannii* persister cells than for *A. baumannii* regular cells. OmpW overexpression plays a key role in the higher number of *G. mellonella* deaths caused by *A. baumannii* persister cells than by *A. baumannii* regular cells. Our research findings OmpW overexpression in meropenem-exposed *A. baumannii* persister cells enhances virulence and reveals a candidate target for preventing and controlling *A. baumannii* persister cells. It is essential to elucidate the pathogenesis of persister cell formation through further research, as well as the putative mechanism for regulating OmpW, which can also further facilitate the development of new vaccines and therapeutic antibiotics.

## Data Availability

The original contributions presented in the study are included in the article/supplementary material, further inquiries can be directed to the corresponding author.
